# Genetic analysis of the modern Australian labradoodle dog breed reveals an excess of the poodle genome

**DOI:** 10.1371/journal.pgen.1008956

**Published:** 2020-09-10

**Authors:** Muhammad Basil Ali, Jacquelyn M. Evans, Heidi G. Parker, Jaemin Kim, Susan Pearce-Kelling, D. Thad Whitaker, Jocelyn Plassais, Qaiser M. Khan, Elaine A. Ostrander

**Affiliations:** 1 Cancer Genetics and Comparative Genomics Branch, National Human Genome Research Institute, National Institutes of Health, Bethesda MD, United States of America; 2 National Institute for Biotechnology and Genetic Engineering (NIBGE), Jhang Road Faisalabad, Punjab, Pakistan; 3 Pakistan Institute of Engineering and Applied Sciences (PIEAS), Islamabad, Punjab, Pakistan; 4 OptiGen, LLC Cornell Business and Technology Park, Ithaca, NY, United States of America; HudsonAlpha Institute for Biotechnology, UNITED STATES

## Abstract

The genomic diversity of the domestic dog is an invaluable resource for advancing understanding of mammalian biology, evolutionary biology, morphologic variation, and behavior. There are approximately 350 recognized breeds in the world today, many established through hybridization and selection followed by intense breeding programs aimed at retaining or enhancing specific traits. As a result, many breeds suffer from an excess of particular diseases, one of many factors leading to the recent trend of “designer breed” development, i.e. crossing purebred dogs from existing breeds in the hope that offspring will be enriched for desired traits and characteristics of the parental breeds. We used a dense panel of 150,106 SNPs to analyze the population structure of the Australian labradoodle (ALBD), to understand how such breeds are developed. Haplotype and admixture analyses show that breeds other than the poodle (POOD) and Labrador retriever (LAB) contributed to ALBD formation, but that the breed is, at the genetic level, predominantly POOD, with all small and large varieties contributing to its construction. Allele frequency analysis reveals that the breed is enhanced for variants associated with a poodle-like coat, which is perceived by breeders to have an association with hypoallergenicity. We observed little enhancement for LAB-specific alleles. This study provides a blueprint for understanding how dog breeds are formed, highlighting the limited scope of desired traits in defining new breeds.

## Author summary

Due to the selective breeding practices used to create modern breeds, dogs suffer from an unacceptable excess of disease, one of several reasons behind the recent trend of creating “designer breeds.” Such populations result from crosses between established breeds, with a goal of producing dogs that lack or have a reduced incidence of unfavorable disorders or phenotypes, yet retain desirable traits from parental breeds. One such example is the Australian labradoodle which was initiated from Labrador retriever and standard poodle crosses 31 years ago in order to produce dogs with a reduced tendency to elicit an allergic reaction but that retained the desirable characteristics of a service dog. In this study we have used nuclear DNA markers to determine the genetic history of the Australian labradoodle and develop an understanding of how breeds stabilize traits given particular breeding strategies. Our data shows that despite the major contributions from poodle and Labrador retriever, additional breeds contributed to the modern Australian labradoodle. Today’s Australian labradoodle is largely poodle with an excess of poodle alleles related to coat type. This study demonstrates that changes in very few genes can define a new breed and demonstrates how breeds can form in a small number of generations.

## Introduction

Approximately 350 domestic dog breeds are recognized by organizations such as the American Kennel Club (AKC) [1] or the Fédération Cynologique Internationale (FCI) [2]. Many other national registries exist to track regional breeds. Integral to the formation of breeds has been the adoption of the breed barrier rule which states that, with rare exceptions, no dog may become a registered member of a breed unless both its dam and sire are similarly registered. Most breeds were established during Victorian times [1, 2], and many are derived from exceptionally small numbers of founders or reflect the use of popular sires, i.e. stud dogs whose gene pools are overrepresented in the population, often followed by strong phenotypic selection [1–3]. Thus, individual breeds are often characterized by restricted gene pools [4–6], permitting identification of loci controlling breed-defining traits using a modest number of genetic markers rather than whole genome sequencing [7–11].

Breeding programs needed to generate and maintain specific traits often require crossing closely related individuals, hence, some domestic breeds have an excess of specific diseases [12], many of which bear striking similarity to those observed in humans [13–16]. In response to these health issues and coupled with a desire to create new breeds with specific attributes, there has been a recent explosion of designer breeds, developed by crossing breeds with specific traits. The resultant lineages are touted as unusually healthy, although for some designer breeds this is a topic of debate [13].

**Fig 1:**
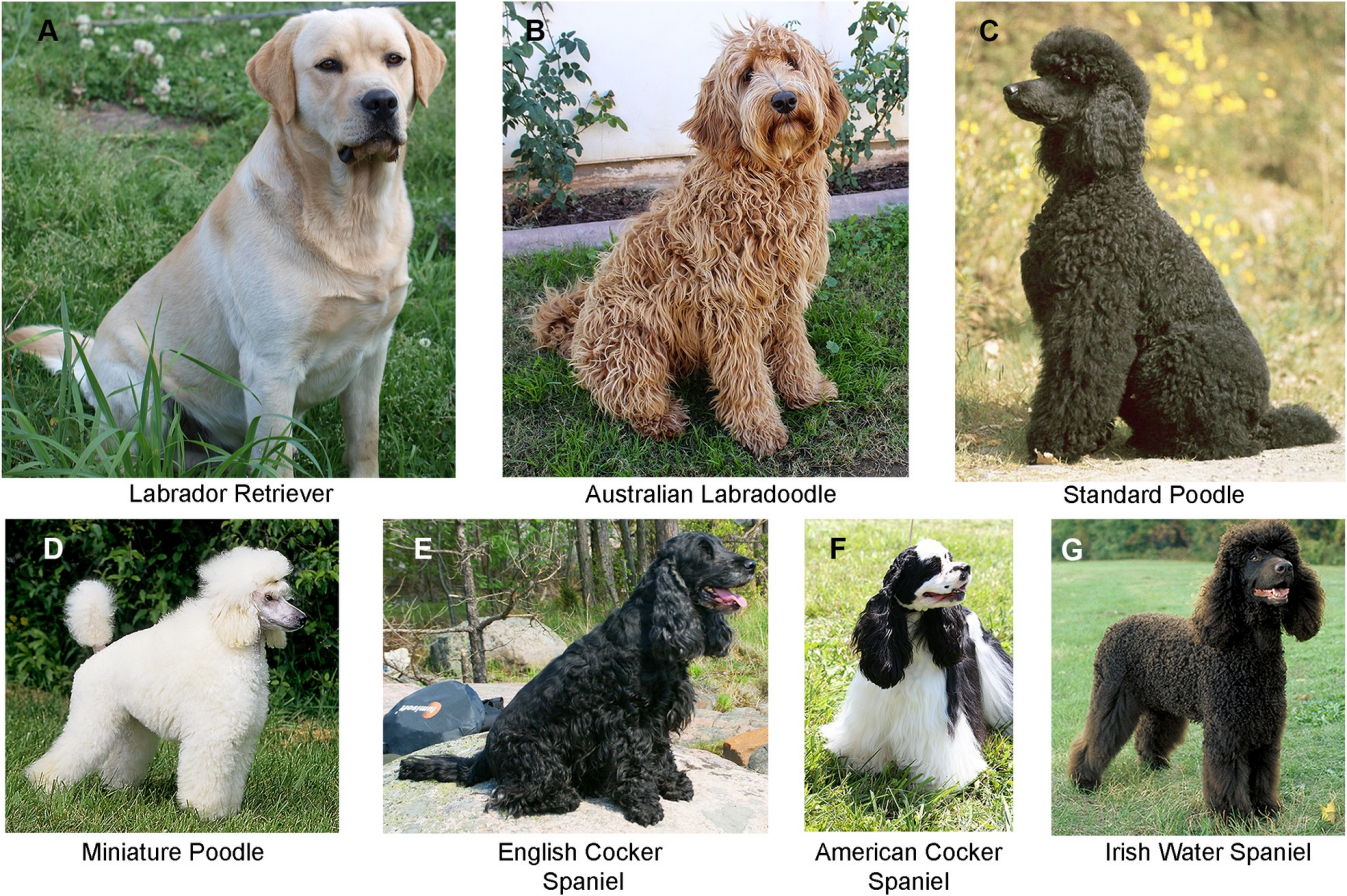
Pictures of the breeds under consideration in this study. (A) Labrador retriever (LAB), (B) Australian labradoodle (ALBD), (C) standard poodle (SPOO), (D) miniature poodle (MPOO), (E) English cocker spaniel (ECKR), (F) American cocker spaniel (ACKR) and (G) Irish water spaniel (IWSP). When all poodle breeds were considered as one group the designation POOD was used.

One of the most popular of the designer breeds is the labradoodle, a cross between the Labrador retriever (LAB) and standard poodle (SPOO) (Fig 1) (https://alaa-labradoodles.com). Today, the name labradoodle describes two populations. The first are F1 hybrids (LBD), which are simply the first-generation product of any LAB and SPOO cross. The Australian labradoodle breed (ALBD), which originated in 1989, is distinct from such hybrids. While also starting as a LAB and SPOO cross, it has since been propagated by planned crosses to establish a true-breeding population, meeting an established set of standards. With an original goal of producing a low-shedding service dog, offspring of the planned crosses are known today as Australian labradoodles, and fanciers seek to have it recognized by an international registry as a unique breed.

In this study we sought to understand how the ALBD breed was created by doing a whole genome analysis of multiple generations. We were specifically interested in determining how breed-defining traits would appear and stabilize given the breeding programs employed. We have used haplotype sharing and admixture analysis to identify constituent breeds. We identify parental breed-associated loci and variants that most strongly distinguish the ALBD from its founder breeds. Finally, an examination of linkage disequilibrium (LD) decay in multi-generational ALBD versus LBD provides insight as to the stability of a quickly developed breed.

## Results

### Breed structure, admixture, and haplotype analyses

**Fig 2:**
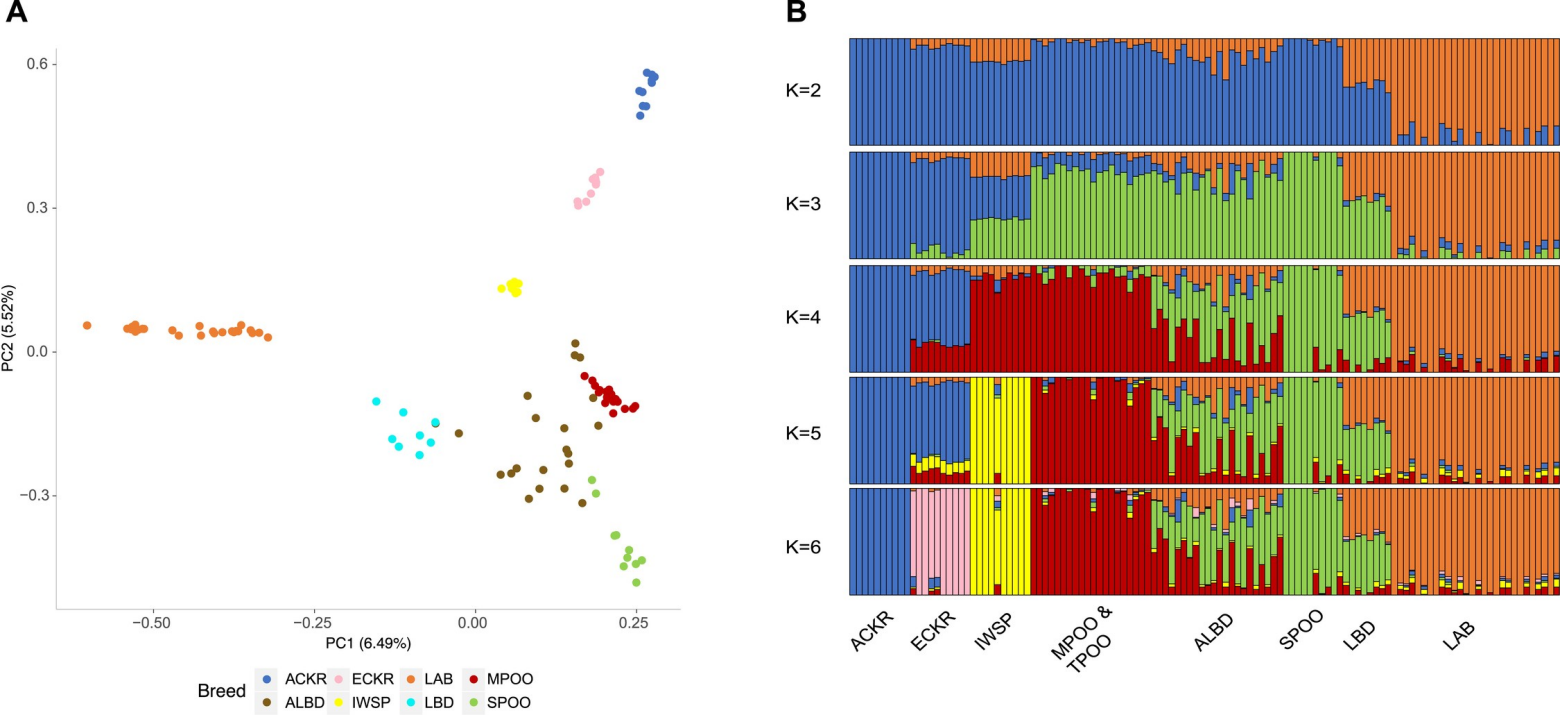
PCA and Admixture analyses. A) PCA analysis plot of the top two principal components (PC1 and PC2), showing the distribution of the eight breeds of interest accounting for a genetic variance of 6.5% and 5.5%, respectively. (B). Admixture analysis representing data for K = 2–6 cluster-based ancestry models. Each bar represents an individual dog, and the color length for each bar represents the proportion of genomic contribution from the founder population.

We initially examined the relationship of the ALBD and LBD to six putative parental breeds using Principal Components Analysis (PCA) (Fig 2A). The first two PCs explain 6.49%, and 5.52% of the total genetic variance observed among breeds, respectively. PC1 provides major separation of the LAB from all other breeds. The central positioning of the LBD between the LAB and SPOO validates the reported background of these samples as F1 hybrids. The second PC separates the POOD, including the SPOO and miniature (MPOO) and toy poodles (TPOO) from a group that includes the English cocker spaniel (ECKR), American cocker spaniel (ACKR), and Irish water spaniel (IWSP), all breeds reported to have been included at least once in ALBD pedigrees (https://alaa-labradoodles.com). Of note, no IWSP was present in the pedigrees of ALBD we tested. The three spaniel breeds clearly separate from the LAB in PC1, as well as from the POOD and each other in PC2. There is overlap between the ALBD and POOD varieties but not with the distantly positioned LAB. These results provide the first indication that the ALBD retains more POOD ancestry than LBD, and both the SPOO and smaller POOD varieties contributed to the breed.

We next estimated admixture in the ALBD and related breeds using a cluster-based ancestry method that applies different models of population structure to the data (Fig 2B). Dividing the dataset into two populations, at K = 2 the LAB separates from the rest of the breeds, and at K = 4 the 50% contribution of SPOO to LBD becomes clear. At K = 4–6 it becomes increasingly evident that the MPOO/TPOO and SPOO are the major contributors to the existing ALBD, as the breed retains only a small contribution from the LAB. There is <5% contribution from the MPOO/TPOO to the LBD, and that is likely to represent the close relationship of the POOD varieties. As each column represents a single dog, the graph shows variation between individual ALBDs, particularly in terms of which POOD variety contributed to each individual, reflecting the still early generations of the population (all dogs <9 generations since the last purebred outcross). The IWSP does not appear to make a contribution to the ALBD based on this dataset. The ECKR and ACKR, which are themselves closely related [1, 17], contribute <5% to a subset of ALBD lineages and none to the rest.

**Fig 3:**
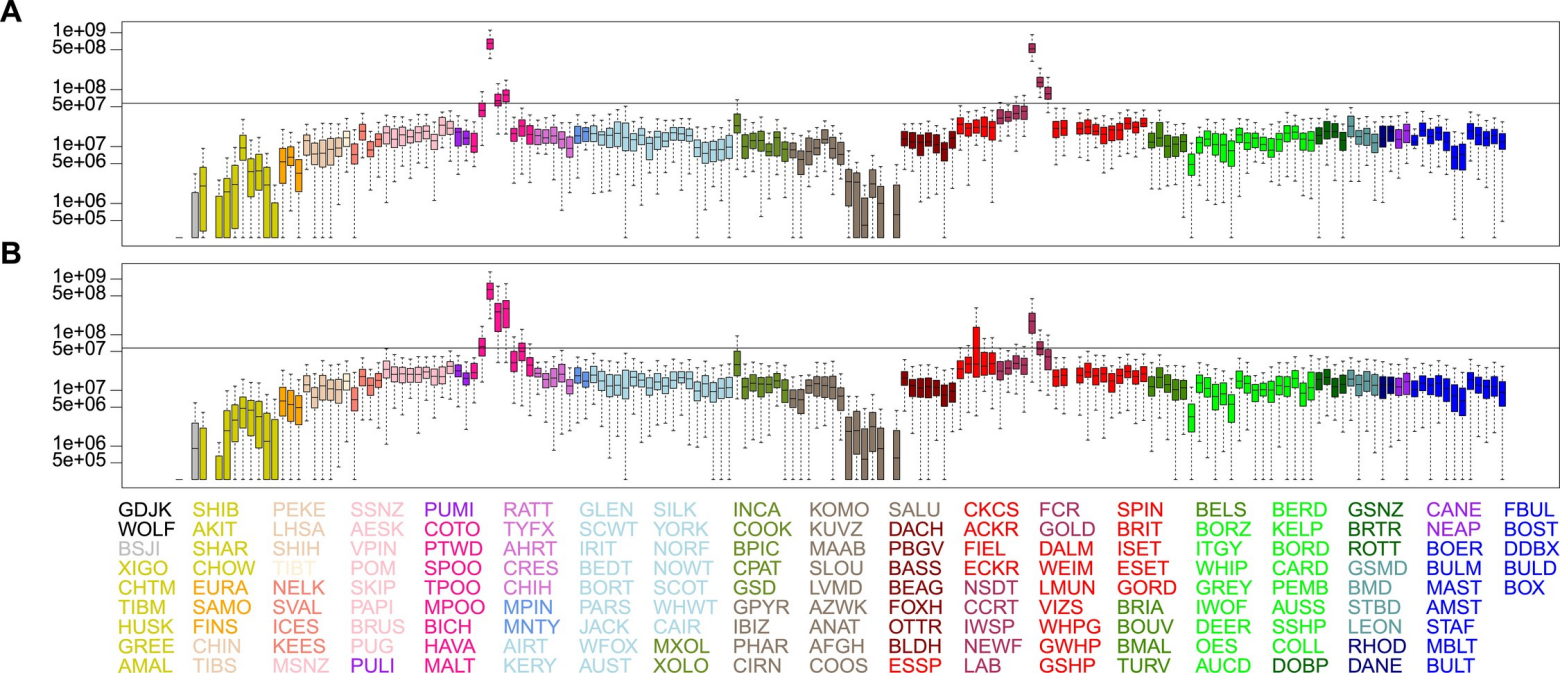
Haplotype sharing analyses of 161 dog breeds including the LBD and ALBD. Haplotype comparison analyses for both LBD (A) and ALBD (B) with 23 domestic dog breed clades, colored separately, are shown. Analysis used a window size of 1900 SNPS with an overlap of 50 SNPs. Breeds are listed and colored on the x-axis. Colors correspond to the 23 previously reported dog breed clades. The y-axis indicates combined haplotype sharing. Breed abbreviations can be found in S2 Table. A significance level of 95% of all across breed sharing is indicated by the horizontal line. Pairs which did not share a haplotype were set at 250,000 bp for graphing.

**Fig 4:**
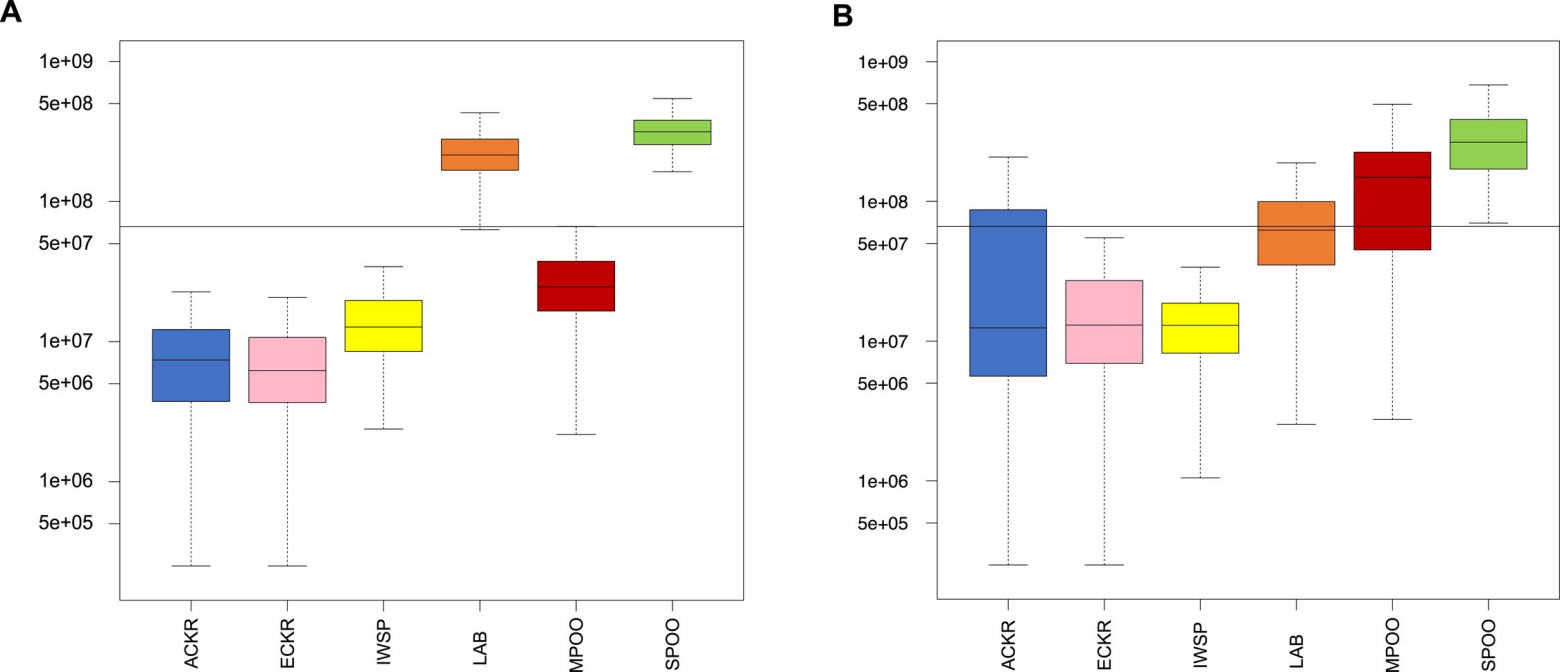
Haplotype sharing analyses of six dog breeds, including the LBD and ALBD. Haplotype sharing boxplot for LBD (A) and ALBD (B), and six component breeds established using a window size of 1,400 SNPs and an overlap of 50 SNPs. The colors of six breeds correspond to the Fig 1. Y-axis indicates total haplotype sharing. Pairs which did not share a haplotype were set at 250,000 bp for graphing.

To further understand the genetic makeup of the LBD and ALBD identity-by-descent (IBD) haplotype sharing between 161 previously published breeds and nine wild canids versus LBD and ALBD was calculated, and two plots were drawn by subsetting LBD (Fig 3A) and ALBD (Fig 3B) [4]. The LAB and SPOO represent the most significant haplotype sharing with the LBD, as expected. Analysis of the ALBD reveals greater haplotype sharing with all three varieties of POOD and the ACKR (Fig 3B). Haplotype sharing with the Havanese, golden- and flat-coated retrievers, and Portuguese water dog is noted for both the ALBD and LBD, potentially reflecting the historical relatedness of those breeds to the LAB (golden and flat-coated retrievers) and POOD (Havanese and Portuguese water dog) and not necessarily admixture with labradoodle populations [4]. Similar results were found in the second dataset, which compared the LBD (Fig 4A) and ALBD (Fig 4B) to the subset of predicted parental breeds. We note, however, that in this reduced dataset the contribution from the ACKR breed crosses the significance threshold, while the contribution of neither of the other two spaniel breeds approaches significance. The proportion of haplotype sharing representing the contribution of LAB to ALBD (0.13) is approximately one-third that of LAB to the LBD (0.38).

**Fig 5:**
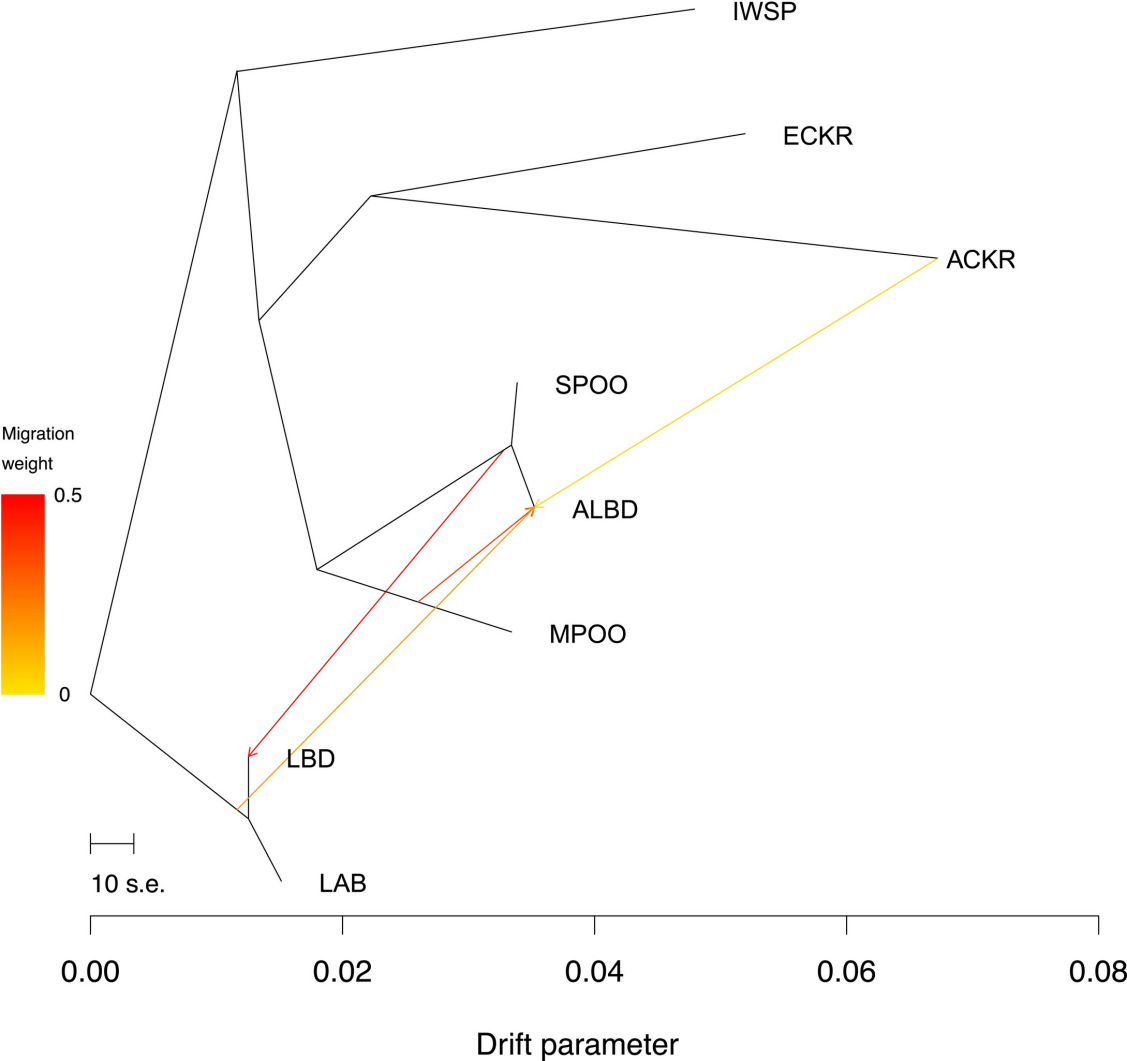
Maximum likelihood tree. Tree was constructed with TreeMix using four migration events, LD, and a bootstrapped over 1,000 bp windows. Breed abbreviations correspond to S2 Table. X-axis indicates the amount of genetic drift. The arrows denote migration from origin to recipient breed, and the weight of migration is scaled according to percentage mixture indicated by the heat map on the y-axis.

As a complement to the above studies, we conducted a TreeMix analysis [18] (Fig 5). TreeMix generates a maximum likelihood tree from a large number of SNPs, producing an estimate of the historical relationships of a set of populations, accounting for both splits in the population as well as migration events. We observe that the LBD and LAB are on the same branch, while the ALBD is found on the POOD branch. Admixture from the SPOO to the LBD is observed at 49%. The contribution of the smaller poodle varieties (combined MPOO and TPOO) to the ALBD is substantial but undetected in the LBD.

To better understand the placement of LBD with LAB, we generated a separate tree using the six established breeds but not the LBD or ALBD (Residual value = -1.4 to 1.4) (S1 Fig.). We observe that the LAB is on a branch with the IWSP, and the SPOO is on a branch with the MPOO, similar to the base tree in Fig 5. We also built a tree including the mixed population, but did not allow for migration (Residual value = -21.9 to 21.9) (S2 Fig.). On this tree, the LAB and SPOO all stem from one branch, suggesting that the LAB and SPOO are closely related, which we know is not true based on the first tree and published phylogenetic studies. When we consider the residuals from this model they show that the LAB and SPOO are positioned too close for this data and the IWSP and LAB are too distant from each other. Thus, the best arrangement for the LBD is on a branch with the LAB, placed distant from the POOD and its relatives, but with migration from the SPOO as shown (Fig 5).

### Inbreeding coefficient analysis

We next examined inbreeding in the ALBD and associated breeds (Table 1). Of the breeds tested, the LBD and the ALBD have the lowest mean inbreeding coefficients (-0.06 and 0.05, respectively), indicating relatively high genetic diversity within each population compared to purebreds. There are multiple explanations for the diversity observed among ALBDs. First, they are mixed breed dogs and the ALBDs included in this work are multigenerational, reflecting a mixture of dogs and breeding schemes. In addition, the primary parental breeds are themselves popular, with large memberships [1] who likely contribute to the overall increase in genomic heterozygosity [19, 20]. Indeed, the potential component breeds present an ordered slope with the SPOO having the lowest level of inbreeding and the ACKR, a breed that diverged from the ECKR in the 20^th^ century [1, 21], having the highest.

**Table 1:** Mean inbreeding coefficient values for ALBD and LBD vs. established breeds.

Breed name	Mean inbreeding coefficient	Min.	Max.
LBD	-0.06	-0.08	-0.04
ALBD	0.05	-0.06	0.2
SPOO	0.12	0.01	0.2
LAB	0.19	0.08	0.26
MPOO	0.19	0.05	0.4
IWSP	0.25	0.17	0.35
ECKR	0.31	0.16	0.43
ACKR	0.37	0.3	0.47

### Allelic variation analysis

**Fig 6:**
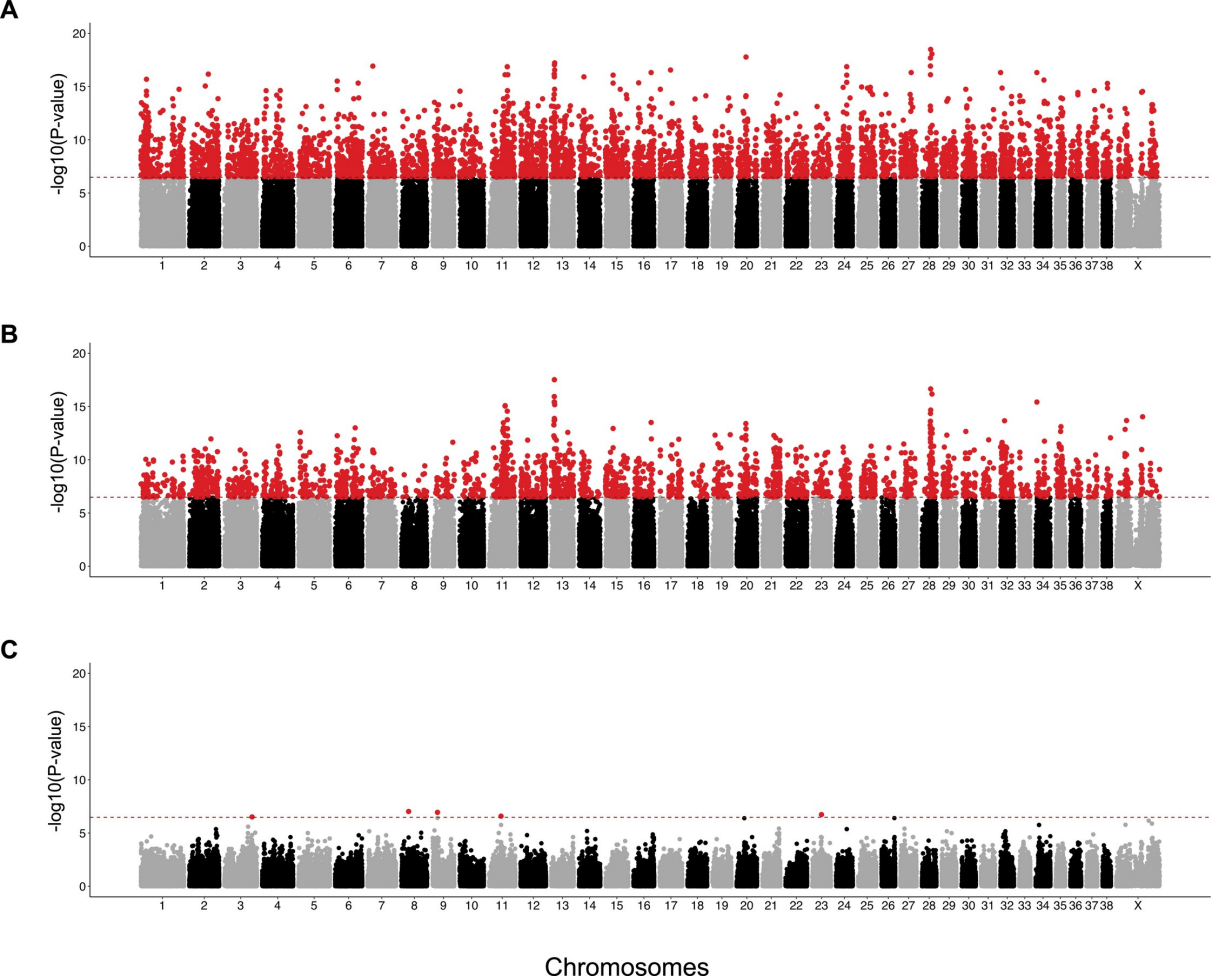
Genome-wide identification of variants with significant allele frequency differences between breeds. (A) Manhattan plot for the POOD vs. LAB allele frequency comparison reveals allele frequency differences on chromosome 13 in a region containing *RSPO2* (peak SNP chr13:8635445) and on chromosome 28 in the region containing *ADRB1-AU1* (peak SNP chr28:24866296). (B) Manhattan plot for ALBD vs. LAB, identifying loci on chromosomes 13 and 28. (C) Manhattan plot for ALBD vs. POOD. Five significant SNPs are noted on chr 3, 8, 9, 11 and 23. The red line represents the Bonferroni corrected significance threshold (-log_10_ (P) = 6.48) and SNPs passing this threshold are colored in red.

To compare allelic variation between the ALBD and its parental breeds we performed three genome-wide comparisons using PLINK [22, 23] (Fig 6). For each comparison, we focused our analyses only on the five most significant SNPs within independent regions. In each case, haplotype blocks surrounding the lead SNP were defined using Haploview [24] by using the solid spine block definition on the basis of LD score. All genes within each haplotype block are reported (Table 2). In the first analysis we compared 20 POOD (10 SPOO, five MPOO, and five TPOO) versus 28 LABs. We observed 6,181 SNPs crossing the Bonferroni threshold (P = 3.33x10^-7^) with genome-wide significant differences in allele frequency distributed across all chromosomes (Fig 6A). Peak SNPs were located in regions containing known genes associated with coat phenotypes, including *r-spondin 2* (*RSPO2*), *keratin 71* (*KRT71*), a lncRNA upstream of *adrenoceptro beta 1*, *ADRB1 (ADRB1-AU1)*, and *c10orf118* [25–27]. The latter is involved in atopic dermatitis in dogs with a dense undercoat [27–29]. Other genes identified in this analysis with significant p-values include *tudor domain containing 1* (*TDRD1*), which plays a role in embryogenesis and gametogenesis [30] and *rab11 family interacting protein 2* (*RAB11FIP2*), which encodes a GTP binding protein [31].

**Table 2:** Top five SNPs with significant allele frequency differences between ALBD and LAB or POODs and most proximal genes.

Breed comparison	Lead SNP	P-value	Allele frequency ALBD	Allele frequency POOD	Allele frequency LAB	Region defined by Haploview	# of SNPs within region	Genes within region
ALBD x LAB	chr13:8635446	3.03e-18	0.86	1	0	8472061–8718212	4	*RSPO2*
ALBD x LAB	chr13:8637613	3.03e-18	0.86	1	0	8472061–8718212	4	*RSPO2*
ALBD x LAB	chr28:24866296	2.22e-17	0.86	0.93	0.02	24814135–25035686	3	*ADRB1-AU1*
								*c10orf118*
								*TDRD1*
ALBD x LAB	chr28:28503227	6.72e-17	0.89	0.95	0.03	28368822–28582831	10	*RAB11FIP2*
ALBD x LAB	chr13:8357088	1.16e-16	0.83	0.90	0.01	8214281–8442632	11	*ANGPT1*
ALBD x POOD	chr8:20741929	9.49e-08	0.64	0.93	0.28	20625037–20989649	17	N/A
ALBD x POOD	chr9:13481554	1.14e-07	0.79	0.8	0.18	13461625–13512270	4	*CACNG4*
ALBD x POOD	chr23:25770325	1.86e-07	0.9	0.65	0.3	25744912–25784450	2	*TBC1D5*
ALBD x POOD	chr11:33845851	2.60e-07	0.62	0.93	0.13	33834431–33924682	4	*MPDZ*
ALBD x POOD	chr3:78254614	3.08e-07	0.86	0.7	0.13	78230838–78274849	2	N/A

We next performed an allelic comparison of 21 ALBDs versus 28 LABs and observed 3,181 SNPs that crossed the Bonferroni threshold, demonstrating significant differences between the ALBD and LAB allele frequencies (Fig 6B). The five most significant SNPs (range P = 10^−16^–10^−18^) are present in the regions containing *RSPO2*, *ADRB1-AU1*, *c10orf118*, *TDRD1*, *RAB11FIP2*, and *angiopoietin 1* (*ANGPT1*). The first five genes were also observed in the POOD vs. LAB comparison, while the last plays a role in vascular development and has been associated with hypoxia adaptation in grey wolves (Table 2*)*. The third analysis compared ALBD to the POOD. In this case, we observed only five SNPs in total passing the Bonferroni threshold, with p-values of 10^−7^–10^−8^ (Fig 6C). Haplotypes were again constructed around each peak, highlighting only three genes: *calcium voltage-gated channel auxillary subunit gamma 4* (*CACNG4*), *TBD1 domain family member 5* (*TBC1D5*), and *multiple PDZ domain crumbs cell polarity complex component* (*MPDZ*). Among these, *CACNG4* is perhaps the most interesting because of its role in the oxytocin signaling pathway.

The large number of significant SNPs observed when comparing POOD to LAB and ALBD to LAB, versus the small number observed when comparing the ALBD and POOD, indicates a much stronger contribution of POOD varieties than the LAB to the ALBD. When examining haplotypes around the lead SNPs we were unable to identify common haplotypes between the ALBD and either parent breed except in regions surrounding *RSPO2* and *ADRB1-AU1*, where the ALBD shared common haplotypes with POOD.

### LD analysis

**Fig 7:**
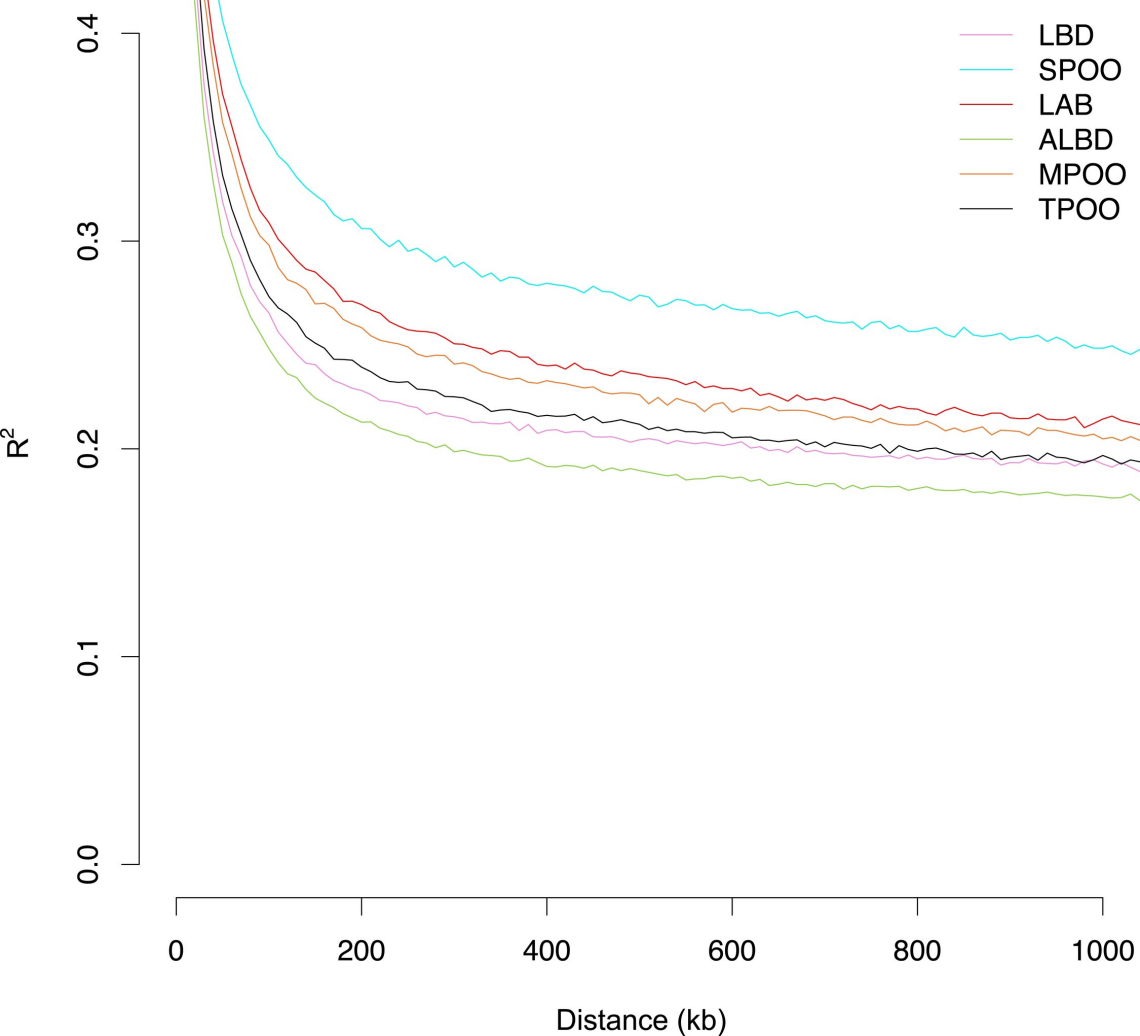
LD decay curve of LBD and ALBD and component breeds. Multigenerational ALBD and LBD decay plots using six dogs per breed are shown in comparison to parent breeds. Data are based on a mean r^2^ using a window size of one Mb.

We next measured the extent of LD in ALBD in comparison to LAB, POOD, and LBD (Fig 7). At one megabase (Mb), LD was most extensive in the SPOO (mean r^2^ = 0.25) and least in the ALBD (mean r^2^ = 0.18). Among purebred dogs, the lowest LD at one Mb was observed in the TPOO (mean r^2^ = 0.20), which is very similar to the LD decay observed by Boyko et al. in a study of 59 domestic breeds [8]. The rapid pattern of decay in ALBD illustrates the high genetic diversity of this breed, showing that LD does not extend very far in their genome. Given the young status of this breed, this is presumably explained by the high degree of hybridization in the creation of the ALBD, which is also reflected in our other analyses. Thus, this pattern of decay explains why we were unable to identify large shared haplotypes around the most significant SNPs in the allele frequency analysis, with the exception of the *RSPO2* and *ADRB1-AU1* regions. Unsurprisingly, LD in the LBD was higher than in the ALBD, as the former comprises the complete genomes of two distinct breeds while the latter reflects some generations of recombination.

### Candidate gene analysis

Since haplotypes across genes associated with coat variation are primarily shared by the ALBD and POOD, and absent in the LAB, we genotyped presumed functional variants in genes associated with coat in the POOD, including coat length (*fibroblast growth factor 5*, *FGF5*), curl (*KRT71*), and furnishings, the latter of which describes the presence or absence of moustache and eyebrows (*RSPO2*) [25, 32, 33]. In addition, we examined two previously reported SNPs located in the intron of a lncRNA upstream of *ADRB1 (ADRB1-AU1)* that is associated with the presence or absence of an undercoat [26] which were also significant in our allelic analysis.

As we have previously reported an association between a 167 bp indel in the 3' untranslated region of *RSPO2* with furnishings [25], we directly genotyped the insertion in ALBDs (Table 3). Fifteen of 21 ALBD were homozygous for the insertion and six were heterozygous. All POOD were homozygous for the insertion while all LABs lack the insertion [25]. These results are supported by observed alternate allele frequencies for the lead SNPs of 1.0 and 0.86 for the POOD and ALBD, respectively, and zero for the LAB, the only one of the three breeds which lacks furnishings (Table 3).

**Table 3:** Genotyping results for coat variants in ALBD, POOD, and LAB.

Position	Gene	Ancestral/Derived	Breed	Hom. ancestral	Het.	Hom. derived	Total
chr13:8610419	*RSPO2*	-/ins	ALBD	0	6	15	21
			POOD	0	0	7	7
			LAB	24	0	0	24
chr27:2539211	*KRT71*	C/T	ALBD	5	8	8	21
			POOD	0	6	24	30
			LAB	28	0	0	28
chr28:24860187	*ADRB1-AU1*	C/T	ALBD	1	9	11	21
			POOD	1	6	3	10
			LAB	0	0	24	24
chr28:24870184	*ADRB1-AU1*	G/A	ALBD	0	2	19	21
			POOD	0	1	9	10
			LAB	21	2	1	24
chr32:4509367	*FGF5*	G/T	ALBD	0	2	19	21
			POOD	0	0	7	7
			LAB	23	1	0	24

Fur length was examined by genotyping a strongly associated SNP (chr32.g.4509367G>T; rs851828354) that is located in the first exon of *FGF5*. The variant causes a cysteine to phenylalanine (ENSCAFP00000013040.3:p.Cys95Phe) change in a highly conserved region of the gene [25, 34]. Nineteen of 21 ALBD and all seven POOD were homozygous for the derived allele (long coat), while 23 of 24 LAB were homozygous for the ancestral allele (short coat).

We also genotyped two mutations in *KRT71*, a keratin gene which has been strongly associated with curly coat in many domestic breeds, including the POOD [25]. The first mutation, chr27.g.2539211C>T; rs23373415, is a nonsynonymous (NP_001183958.1p.Arg151Trp) alteration. Our dataset revealed that 24 of 30 POOD and eight of 21 ALBD were homozygous for the derived (curl) allele, and all 24 LABs were homozygous for the ancestral (non-curl) allele. We genotyped a second variant located in the seventh exon of the same gene (chr.1266_1273delinsACA), which causes a frameshift/stop loss, changing the encoded *KRT71* protein sequence (NP_001183958.1; p.Ser422ArgfsTer?) in curly coated retrievers [32, 33]. No ALBD carried the mutation. Overall, this suggests that either curly coat is not a highly selected trait in the ALBD, or that other genes or previously unreported mutations control the phenotype in this breed. Given the location of *KRT71* within a cluster of keratin genes, and the long LD observed across the dog genome, the latter is a formal possibility.

Finally, we genotyped two SNPs (chr28.g.24860187C>T and chr28.g.24870184G>A) within *ADRB1-AU1* [26]. While the first SNP was previously identified as most closely associated with having a single coat [26], we observe here that the derived allele at chr28:24870184 is most common in the POOD and ALBD. No ALBD or POOD were homozygous for the ancestral allele at the latter SNP, although 21 of 24 LABs were. Conversely, 19 of 21 ALBD and nine of 10 POOD were homozygous for the derived allele (Table 3) at the second SNP. LABs have a double coat which is comprised of a dense undercoat of short hairs and a topcoat of longer “guard” hairs. POOD are reported to have single coats [35], although this is not part of their breed standard; therefore, they were not included in the initial study of single versus double-coated breeds [26]. Double coats in the ALBD are considered a fault by the Australian Labradoodle Association of America, but it is not part of the standard. Thus, while this SNP appears to be related to presence or absence of undercoat, we cannot formally eliminate the possibility that it is a marker for another trait.

To determine if genes affecting the coat are enriched in the SNPs showing significant allele frequency differences between ALBD and LAB, we performed an intersection analysis between the 3,181 SNPs with significant allele frequency differences between ALBD and LAB versus 146,925 SNPs with no significant difference. This revealed 24 SNPs within 250kbp of 11 genes associated with coat color and 69 SNPs within the same distance of 11 coat growth and texture genes. We compared this to 1,164 and 1,161 SNPs that appear in the coat color and growth/texture regions, respectively, but show no significant allele frequency differences. The Fisher’s exact p-value for an enrichment of coat growth and texture genes was <0.00001, with an odds ratio of 2.78 (95% CI 2.18–3.6). In contrast, the Fisher’s exact p-value for coat color genes was >0.05 with an odds ratio of 0.95 (95% CI 0.63–1.4).

## Discussion

Designer dogs have become popular in recent years reflecting increased owner demand for pets that meet their wishes for specific traits, often related to personability, body size, or hoped-for low allergenicity, while retaining favored traits from familiar breeds. Most designer dogs represent first generation crosses, and there is generally little motivation to develop a hybrid into a true breed as subsequent generations, often produced by crossing closely related individuals, may reveal undesirable, recessively segregating traits. The exception is the ALBD, which began with crosses between LAB and SPOO in the 1980s. The original goal was to create a service dog with high biddability that was suitable for households with allergies or asthma, although whether the breed is in fact hypoallergenic, itself a poorly defined word, is a topic of debate. The resulting dogs are smart, friendly, and widely employed as assistance and therapy dogs. The ALBD is not yet recognized by any international registry, but breeding programs and breed standards are defined by the Australian Labradoodle Association of America (https://alaa-labradoodles.com).

We examined the ALBD genome to understand the genetic origins of the breed, including the reported introduction of at least five breeds in addition to the SPOO and LAB. We used genome-wide SNP data to determine if the modern ALBD achieves the metrics that define a distinct breed such as high inbreeding coefficients. By comparing breeds, we sought to understand how the ALBD has changed since its inception, approximately 10 generations earlier, and to identify traits preferred by breeders. Our data reveal that the modern ALBD is mostly POOD, and the POOD alleles related to coat are present in high frequency in the ALBD.

Our dataset comprised seven populations: the LBD, which is a group of first-generation labradoodle dogs; multigeneration ALBD; all three POOD varieties (miniature, toy, and standard); the LAB; ECKR and ACKR; and IWSP (Fig 1). Based on the limited pedigree data available to us, we expect the ALBD to have retained more POOD than LAB in later generations. Our data unambiguously supported that hypothesis. PCA analysis (Fig 2A) shows that the ALBD is a population closely related to POOD. Also, admixture analysis demonstrates that while the LBD are clearly a 50:50 mix of LAB and SPOO, the multigeneration ALBD dogs tested are largely POOD, retaining little LAB contribution. In addition, unlike the LBD, multiple POOD size varieties may contribute to a single ALBD lineage. We also noted minor contributions from spaniels in some lineages, indicating that they were used infrequently, or that their traits were not highly desired in breeding programs.

Finally, the above conclusions are supported by our haplotype analysis which utilized two distinct datasets: the LBD hybrid versus 161 breeds which we described previously [4], and the ALBD versus the same dataset. While strong contributions from the LAB and POOD are noted, these data highlight a common problem in mixed breed dog analysis: separating historical contributions that lead to the creation of the founder breeds versus introduction of unexpected breeds in a particular pedigree. Specifically, we note haplotype sharing with Havanese as well as golden and flat-coated retrievers for both the ALBD and LBD. However, as shown previously, these breeds are from the same genetic clades as the LAB and POOD. Thus, these events likely reflect breed relatedness within the clade [4].

The rapid LD decay observed in ALBD combined with the low inbreeding coefficients reflect the high genetic diversity of their genome. This observation is also explained by the very young nature of this “breed in formation,” which also provides an explanation for why no particular haplotype structure is observed in genes associated with coat type. Allelic frequency analysis was used to identify loci shared by POOD and ALBD versus LAB. Our control comparison of POOD versus LAB identified thousands of SNPs with significant differences in allele frequency. That the LAB versus ALBD comparison also yielded thousands of significant SNPs, while the ALBD versus POOD yielded only five indicates that ALBD are more similar to POOD than to LAB and likely reflects breeding programs which favor reintroduction of POOD over multiple generations.

There are a wide range of body sizes in the ALBD which meet the breed standard. These are assessed as height at the withers (shoulder) and weight. Miniature varieties are 14–16 inches in height and weigh 15–25 lbs; medium ALBD are 17–20 inches and weigh 30–45 lbs; the standard size ALBD are 21–24 inches tall and weigh 50–65 lbs (https://alaa-labradoodles.com). This variation could reflect the inclusion of all three POOD varieties into ALBD breeding programs, as demonstrated by our admixture and haplotype analyses. However, no SNPs associated with body size loci were among the top five most significant in this analysis. For instance, the *IGF1* locus, which accounts for about 15% of variation in body size in domestic breeds [11] is ranked 2622^nd^ in our allelic comparison study, suggesting that the smaller varieties of POOD are not, at present, highly favored in breeding programs.

We also readily acknowledge that our data cannot distinguish between the putative contributions MPOO and TPOO, but it is an interesting issue for breeders. A recent study of 14 behavior traits assayed in 166 labradoodles using the well-established C-BARQ on-line questionnaire [36, 37] revealed that MPOO had a higher score than the labradoodle for social-fear and separation-related anxieties, both undesirable traits, while the SPOO, by comparison, had a lower score than the MPOO for touch sensitivity, which is regarded as a desirable trait [38]. These differences are interesting and worth consideration in future development of ALBD lines or other designer breeds.

We performed direct testing for variants in *KRT71* that have been associated with curl in many breeds, including the POOD [25]. While we observed the expected distribution of alleles in the POOD and LAB, the genotypes were evenly distributed in the ALBD. This was surprising given our perception of how many ALBD have curly or wavy coats. However, the ALBD breed standard permits coat curl to be anywhere on the continuum from straight to wavy to tightly curled. Our data do not exclude the possibility of other genes with variable or weak alleles playing a role in ALBD coat curl.

Finally, we genotyped a mutation in *FGF5* at chr32:4509367, which has been associated with fur length [25, 34]. We observe small differences between the POOD, in which 100% of dogs were homozygous for the derived allele (long hair), versus the ALBD, for which 90.5% were homozygous and 9.5% were heterozygous. Finally, all but one LAB was homozygous for the ancestral (short hair) allele (Table 3). While this again is supported by the ALBD breed standard which calls for fur up to four inches in length, we did not observe a significant SNP at this locus in the allele frequency analysis. We speculate that the result is due to a lack of tagging SNPs on the array for this mutation in the breeds tested. As we did not have specific fur measurements for each individual, we are unable to validate this. We similarly found no genes associated with coat color among the five most significant SNPs. However, the tagging SNPs for *melanocortin 1 receptor* (*MC1R*) and *agouti signaling protein* (*ASIP*) ranked 2,738^th^ and 2,904^th^, respectively, in the allele frequency comparison, indicating some skewing of coat color alleles in the ALBD versus parental breeds. This is consistent with the breed standard, as the Australian Labradoodle Association of America accepts 14 coat colors including variants of cream, apricot, silver, red, chalk, and black; it also reflects the degree of coat color variation observed in the POOD but not the LAB.

Low shedding, tight coats, and lack of a double coat are perceived as key traits contributing to the perceived “hypoallergenic” nature of established breeds like the Bichon Frisé, Portuguese water dog, and POOD. Indeed, POOD have been the breed of choice for the creation of many designer crosses such as the yorkipoo, peekaboo, lhasapoo and pomapoo. The intellect of the POOD combined with a desirable coat and ease of trainability are all traits which make the POOD a desirable inclusion in a hybrid. Many people with dog allergies do not have an allergic response when in proximity to breeds that share coat traits with the POOD, including the Portuguese water dog and Bichon Frisé. We note, though, that while there is some anecdotal evidence, a rigorous survey regarding the allergenicity of designer breeds has not, to our knowledge, been done. Our results, highlighting the coat and skin genes *RSPO2*, *ADRB1-AU1*, *FGF5*, and *c10orf118*, do not exclude the possibility of other genes, such as those controlling shedding and inherent immune factors, playing a role in the reduced allergic response to the ALBD that has been reported.

Three additional loci which include the genes *CACNG4*, *MPDZ*, and *TBC1D5* were evident in our comparison of ALBD versus POOD (Table 2). None are obvious candidates contributing to known dog traits; rather, all play roles in basic cell biology. *CACNG4*, for instance, is a calcium channel regulator with many roles, among the most interesting is its involvement in the oxytocin signaling pathway, potentially suggesting a role in behavior [39]. Given the different personalities of the POOD and LAB breeds it would be interesting to investigate this finding in greater detail [39]. *TBC1D5* regulates endosomal trafficking [40], and *MPDZ* is related to eye and brain anomalies [41, 42]. We note, however, that the analysis here compared allele frequency only and examined a window-size of 39–364 kb based on shared LD within breeds. Thus, these genes may serve as markers of other adjacent genes outside of the window which are minor contributors to the ALBD.

In a recent podcast with Australian NBC News, Wally Conron, a developer of the ALBD stated publicly that he wished he had not created the breed, and that he had “opened a Pandora’s box” and released “a Frankenstein monster,” a comment made in relation to health issues associated with unscrupulous breeding practices [43]. He and others have argued that designer breeding programs have the potential to reveal recessive traits which accentuate latent genetic health issues [12, 15]. “Hybrid vigor” does not always prevail, particularly if parental breeds share common disease causing alleles [44, 45]. Patellar luxation, Von Willebrand blood clotting disease, progressive retinal atrophy, and hip and elbow dysplasia are common health problems for the ALBD. Given the popularity of the breed, these issues should be addressed. Our study demonstrates the overwhelming POOD component of the ALBD, providing a direction for the development of genetic tests to be incorporated into thoughtful breeding programs designed to enhance the attributes of this designer breed. This knowledge, in turn, will contribute to the growing body of data from man’s best friend with the potential to enhance our understanding of human health.

## Materials and methods

### Ethics

All protocols were approved by the National Human Genome Research Institute (NHGRI) Animal Care and Use Committee as per protocol GFS-05-. Dog owners provide written consent for sample collection.

### Samples

Published genotype data from ten LAB; 10 each of MPOO, TPOO and SPOO; and 10 each of ACKR, ECKR, and IWSP were used in these analyses [4]. Breeds were selected for inclusion based on both known and reported historical contribution to the ALBD. In addition to the above, 18 LABs were genotyped and added to the dataset. Of the 28 total LABs, 17 were female and 11 were male. Fifteen of the 28 were from a range of geographically distant U.S. states and 13 were of unknown geographic origin. The POOD data included 10 SPOO: five males and five females. Four MPOO were males, and six were females; all were collected from a variety of U.S. geographic areas with the exception of one from Ontario, Canada. The 10 TPOO included five males and five females, all of which were from distributed US geographic states. The 10 ACKR, ECKR and IWSP included five males and five females each. All DNA samples from these breeds are from AKC registered dogs that were, to the best of our knowledge, unrelated to at least the grandparent level.

DNA from 21 ALBD, nine females and 12 males, and eight LBD, the latter of which were first generation LAB/POOD crosses, was provided by Optigen, LLC (Ithaca, NY). The pedigrees provided for the ALBD are sufficiently complex that naming generations is difficult. Thus, for this ALBD analysis, generations were numbered back to the most recent purebred individual in the pedigree, up to four generations. Dogs with more extensive pedigrees were termed 4+. The “multigenerational” descriptor indicates the complex nature of the ALBD pedigrees. Three ALBD were of uncertain generation but were clearly ≥4 generations. To our knowledge, 19 ALBD were unrelated at the grandparent level. Of the remaining, there was one set of siblings and a second set of dogs who share one grandparent.

### Genotyping

All DNA samples were genotyped on the Illumina CanineHD SNP chip (San Diego, CA). The SNP dataset for LAB, SPOO, MPOO/TPOO, ACKR, IWSP and ECKR was previously described [4], and had been genotyped for 150,112 SNPs. Genotype calls for the LBD, ALBD and additional 18 LABs were performed using Illumina’s Genome Studio v.2 (Illumina, San Diego, CA). Samples with a >90% SNP call rate were retained. SNPs with Gentrain scores >0.4 and minor allele frequency >1% were retained. Both dataset were merged using PLINK v.1.9 [23]. The final dataset includes 150,106 SNPs.

### Statistical analysis

Principal components (PCs) were calculated using FlashPCA software from PLINK formatted bed files containing 39 chromosomes [46]. We sought to determine the component breeds that went into the construction of the ALBD. While several are reported on web sites or by breeders, these may not be necessarily true and minor breeds may have contributed to a subset of ALBD lineages. While not establishing the relatedness of breeds, like a phylogenetic analysis, admixture analysis can identify contributions from other breeds that happened several generations previously. We utilized ADMIXTURE v1.23, with two to ten adjusted cluster ancestry models [47]. Results are shown for K = 2–6. The optimal K (K = 5) was determined using the cross-validation procedure (S3 Fig.) meaning that based on allele distribution the optimal number of populations in the dataset is five.

IBD haplotype sharing analysis and phasing was performed with BEAGLE v4.1 using variant call format (VCF) files generated with PLINK v1.9 [23], which permitted dissection of ALBD parental breeds [48, 49]. For these analyses, two distinct datasets were constructed. The first contained SNP data from 1,346 domestic dogs representing 161 breeds and nine wild canids genotyped at 150,112 SNPs, as previously reported [4]. The second included data from only the seven breeds outlined above, all of which are hypothesized to have contributed to the ALBD. Dataset one utilized a window size of 1,900 SNPS with an overlapping value of 50 SNPs, while the second set used a window size of 1,400 SNPs, also with an overlapping value of 50 SNPs. A threshold value of 95% was calculated using the quantile function in R based on all possible pairs of dogs from different breeds. This, in turn, was used to determine which breeds contributed to each labradoodle population. Haplotype sharing was considered to be significant between the two populations if the upper quartile of the box is higher than the 95^th^ percentile boundary calculated from all haplotype pairs. The proportion of genomic contribution by the LAB to the ALBD and LBD was calculated by extracting average haplotype sharing of all pairs of LBD or ALBD with LAB and dividing that number by the combined average haplotype sharing of all pairs of LBD or ALBD with ACKR and POOD. The inbreeding coefficient was calculated using the ‘—het’ function of VCFtools v.0.14 [50]. R was used to construct graphs and plots for all analyses [51].

To further evaluate admixture, a maximum likelihood tree was constructed using TreeMix v.1.12 [18]. The tree was constructed using the population-based allele frequencies of approximately 150,106 SNPs. The tree was bootstrapped by analyzing the data in windows of 1000 SNPs using the flags -bootstrap and -k 1000. We repeated the analysis with zero to four migration events allowed and found no further migrations beyond four. The residuals, which indicate how well the data fits the model, showed the lowest range (-1.4 to 1.4) at four migrations. Two additional trees (S1 and S2 Figs.) were constructed using established breeds (with and without the LBD or ALBD) (S1 and S2 Figs.) and the same set of markers and parameters defined above, but with no migration.

### Allele frequency comparisons

Allele frequency comparison analyses were conducted using the—*assoc* function in PLINKv1.9. Three comparisons were as follows: POOD versus LAB, ALBD versus LAB, and ALBD versus POOD. Allele frequency comparisons were done using the 28 LABS described above. We utilize a balanced population of 20 POOD, 10 SPOO and five each of MPOO and TPOO. This included five female and five male SPOO, three female and two male MPOO, and two female and three male TPOO. Haploview [24] was used to establish haplotype boundaries around the top five SNPs from each comparison using the solid spine block definition on the basis of LD score, and excluding SNPs with a minor allele frequency <5%. Manhattan plots were generated using R.

### LD decay analysis

Pairwise LD was summarized using the genotype correlation coefficient r^2^ and decay plots were made using PopLDdecay [52]. For all autosomal SNPs a one Mb window was used. We calculated the r^2^ between all pairs of SNPs (unphased), using PopLDdecay, where both SNPs had a MAF ≥15% and ≤10% missing data. We used a break point of 100,000 bp and *-bin2* flag of 10,000 bp to smooth the lines. To compare LD between breeds, a random set of six individuals from each breed were selected. For ALBD, six dogs, all representative of the multigenerational nature of the designer breed, were selected.

### Candidate gene analysis

All samples were analyzed for putative functional mutations in *FGF5*, *RSPO2*, *KRT71*, and *ADRB1-AU1* [25,32, 33, 26]. Mutations were genotyped using Sanger sequencing (*FGF5*, *KRT71*, *ADRB1-AU1*) or gel band size discrimination (*RSPO2*). Using manufacturer protocols, a touchdown polymerase chain reaction (PCR) protocol with AmpliTaq Gold (ThermoFisher Scientific, Gaithersburg, MD) was used to amplify DNA fragments. The resultant PCR products were purified using Exosap (ThermoFisher Scientific, Gaithersburg, MD), Sanger sequenced using BigDye Terminator v3.1 (Applied Biosystems, Foster City, CA), and analyzed on an ABI 3730xl DNA analyzer (Applied Biosystems, Foster City, CA). Genome positions of all primer sequences are provided in (S1 Table). With the exception of *KRT71* and *ADRB1-AU1* we used a previously published catalog of 722 WGS [11] to determine mutations in the LAB and POOD. The sequence of each primer, name of the relevant gene, primer Tm, and position of each variant is listed in S1 Table.

To determine if genes affecting the coat are enriched in SNPs showing significant allele frequency differences between ALBD and LAB, we made a 2x2 contingency table from the two sets of SNPSs; 3,181 with significant allele frequency differences and 146,925 showing no significant differences. We first counted the number of SNPs that fell within known genes affecting coat traits. The first set comprised coat color genes (*ASIP*, *tyrosinase related protein 1* (*TYRP1*), *melanophilin* (*MLPH*), *MC1R*, *defensin beta 103A* (*DEFB103/CBD103*), *silver locus protein homolog* (*PMEL/SILV*), *MITF*, *solute carrier family 45 member 2* (*SLC45A2*), *proteasome subunit beta type-7* (*PSMB7*), *solute carrier family 2 member 9* (*SLC2A9*), *KIT ligand* (*KITLG*)) while the second comprised coat growth and texture genes (*FGF5*, *RSPO2*, *KRT71*, *melanocortin 5 receptor* (*MC5R*), *ADRB1-AU1*, *forkhead box I3* (*FOXI3*), *serine/threonine-protein kinase 3* (*SGK3*), *fibroblast growth factors 3 and 4* (*FGF3* and *FGF4*, respectively) *FGF5* and *oral cancer overexpressed 1* (*ORAOV1*)). To allow for LD, we increased the total length of each gene by 250kb at both ends. Individual bedfiles were created for gene and SNP loci and the overlapping positions were identified using the intersect function in bedtools v2.29.2. The Fischer’s exact test p-value was calculated by online Fischer test calculator (https://www.socscistatistics.com/tests/fisher/default2.aspx) and the odds ratio was calculated using the online tool (https://www.medcalc.org/calc/odds_ratio.php).

## Supporting information

S1 Fig Maximum likelihood tree. Tree for six established breeds was constructed with TreeMix using a bootstrap value of 1,000. Breed abbreviations correspond to S2 Table. The weight of migration is scaled according to percentage mixture indicated by the heat map on the left.
(TIF)

S2 Fig Maximum likelihood tree. Tree for six established breeds including ALBD and LBD was constructed with TreeMix. Breed abbreviations correspond to S2 Table. The weight of migration is scaled according to percentage mixture indicated by the heat map on the left.
(TIF)

S3 Fig Cross validation (CV) error plot for admixture analysis. Line graph of CV error values for each ancestry models denoted by K.
(TIF)

S1 Table Primer sequences, Melting temperature (Tm), and PCR product size for each genotyped variant. (DOCX)

S2 Table Breed abbreviations. Summary of breed abbreviations corresponding to Fig 3A and 3B.
(DOCX)
