# Supplementary figures and images for: Genetic analysis of the modern Australian labradoodle dog breed reveals an excess of the poodle genome
Source: PLoS Genet. 2020 Sep 10;16(9):e1008956. doi: 10.1371/journal.pgen.1008956 (PMC7482835; doi:10.1371/journal.pgen.1008956)

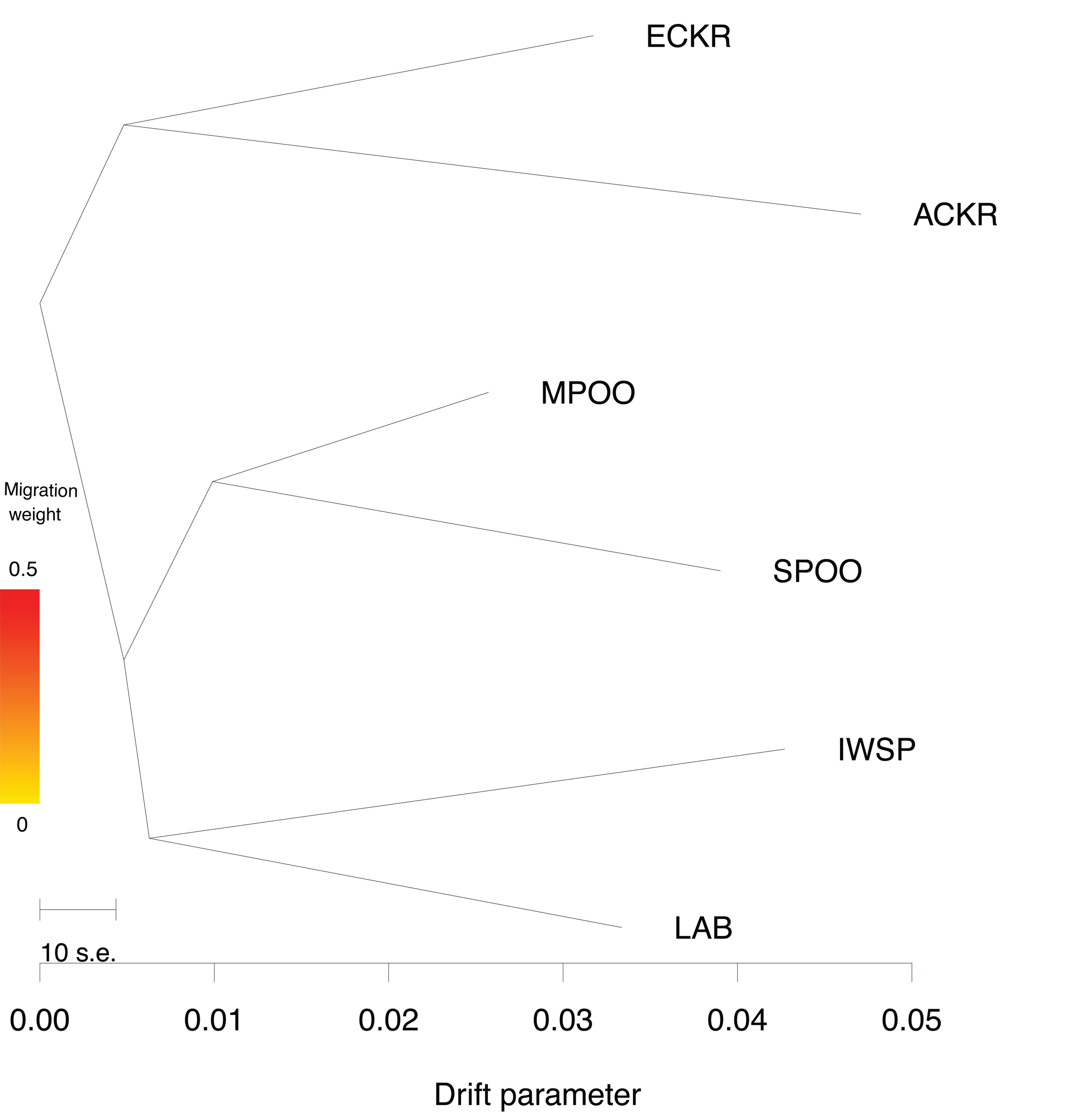

Supplement: S1 Fig — Tree for six established breeds was constructed with TreeMix using a bootstrap value of 1,000. Breed abbreviations correspond to S2 Table. The weight of migration is scaled according to percentage mixture indicated by the heat map on the left. (TIF) [file pgen.1008956.s001.tif]

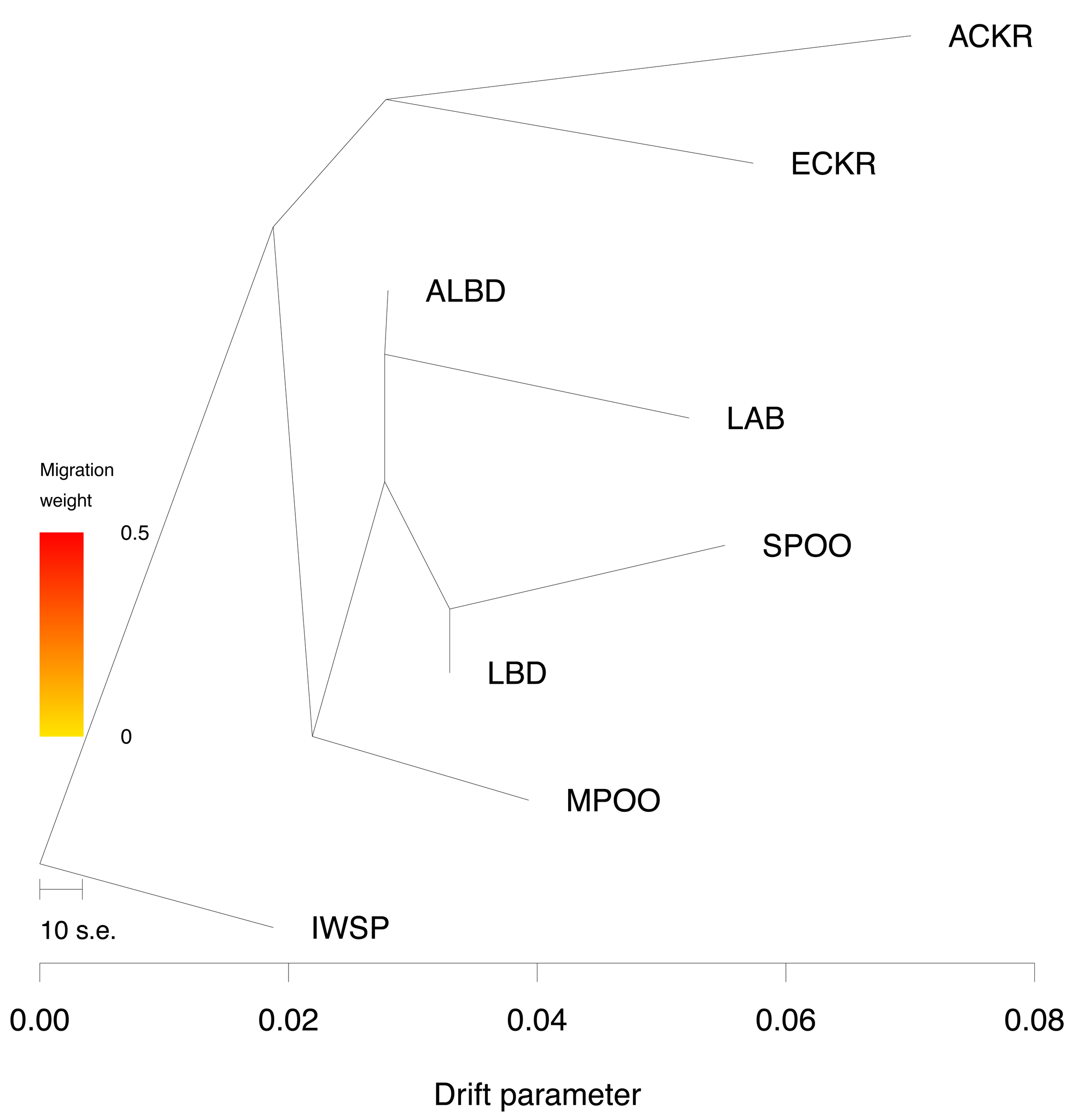

Supplement: S2 Fig — Tree for six established breeds including ALBD and LBD was constructed with TreeMix. Breed abbreviations correspond to S2 Table. The weight of migration is scaled according to percentage mixture indicated by the heat map on the left. (TIF) [file pgen.1008956.s002.tif]

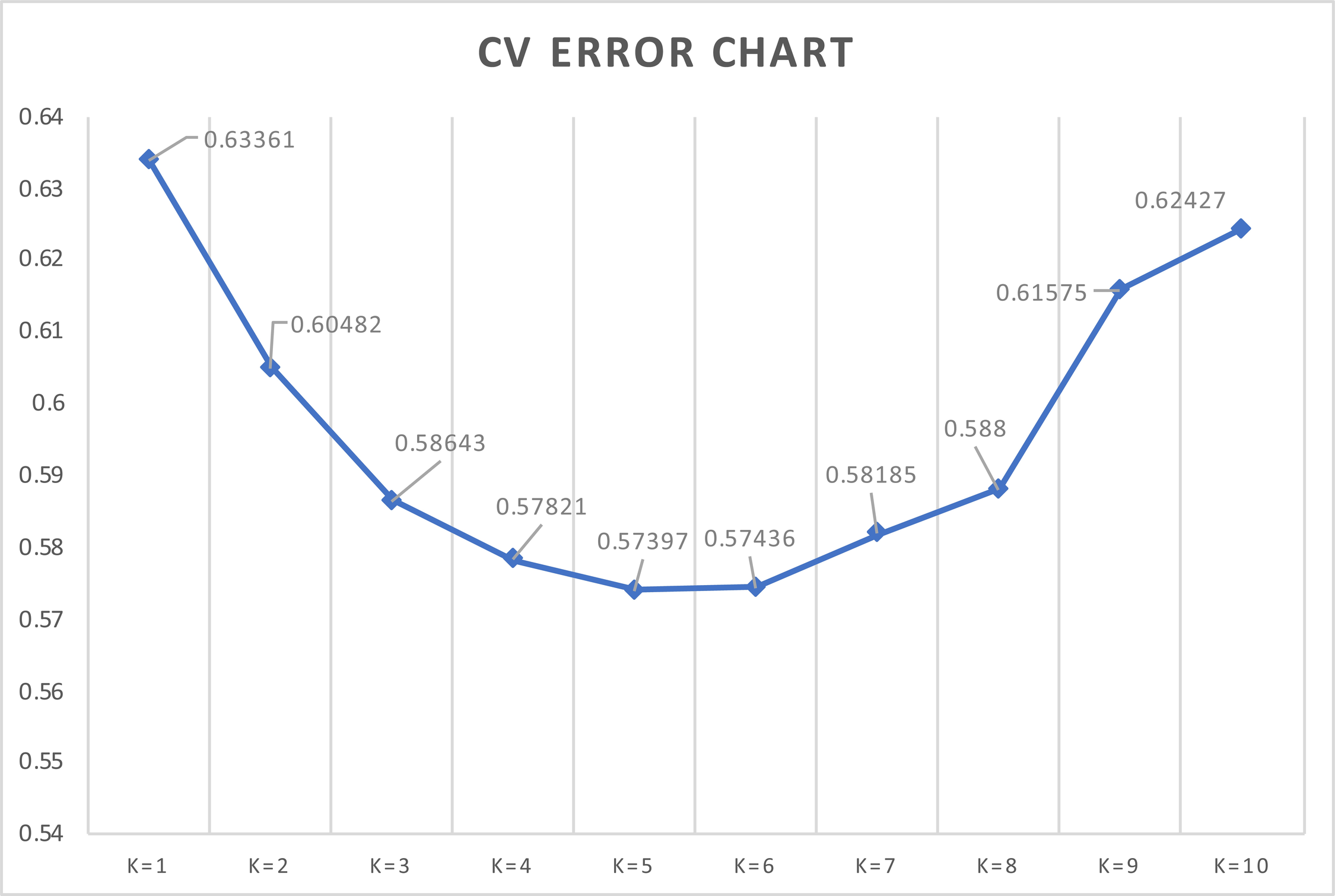

Supplement: S3 Fig — Line graph of CV error values for each ancestry models denoted by K. (TIF) [file pgen.1008956.s003.tif]
