# Supplementary material for: Genetic analysis of the modern Australian labradoodle dog breed reveals an excess of the poodle genome
Source: PLoS Genet. 2020 Sep 10;16(9):e1008956. doi: 10.1371/journal.pgen.1008956 (PMC7482835; doi:10.1371/journal.pgen.1008956)
Supplement: S1 Table — (DOCX) [file pgen.1008956.s004.docx]

**S1 Table.** **Primer sequences, Melting temperature (Tm), and PCR product size for each genotyped mutation.**

| Name of Gene | Primer | Sequence (5'🡪3') | Tm | Product size | Position | Mutation | Method of detection |
| --- | --- | --- | --- | --- | --- | --- | --- |
| *RSPO2* | F^a^ | TGGCTAAAGAAAACTTCCACAA | 58.92 | 676 | chr13:8610419 | 167bp insertion | Gel electrophoresis |
|  | R^b^ | AAATTACCATCATGAGACCATGC | 60.1 |  |  |  |  |
| *FGF5* | F | CTCTCCTCCCCGAGGCTAT | 63.16 | 575 | chr32:4509367 | G>T | Sanger sequencing: ALBD; WGS: LAB and poodle |
|  | R | CCAGGGTGCAAAACAACC | 55.56 |  |  |  |  |
| *KRT71* | F | GTTGTGAGGGGCAAAGGAGAAAATG | 67.7 | 392 | chr27:2539211 | C>T | Sanger sequencing: ALBD, poodle; WGS: LAB |
|  | R | CTGCAGAAGGAGCTCAGAGGGAAAG | 67.9 |  |  |  |  |
| *KRT71* | F | GGAGGTGTGAAGGGAGGAGGTG | 66.54 | 486 | chr27:2543230 | NM_001197029, 1:c.1266_1273delinsACA | Sanger sequencing: ALBD |
|  | R | CCGGACGTATTCTGAGTTACTGTTG | 63.15 |  |  |  |  |
| *ADRB1-AU1* | F | CCACCATCTGTAGCCCTCAT | 59.95 | 523 | chr28:24860187 | C>T | Sanger sequencing: ALBD, poodle; WGS: LAB |
|  | R | TCAGCATGCCACACTTTCTC | 59.99 |  |  |  |  |
| *ADRB1-AU1* | F | CAGGCATTCCTCTCTCTTGG | 59.94 | 521 | chr28:24870184 | G>A | Sanger sequencing: ALBD, poodle; WGS: LAB |
|  | R | GGGCTGGGGATATATTTTGG | 60.35 |  |  |  |  |

^a^F=Forward

^b^R=Reverse
