# Supplementary material for: Genetic analysis of the modern Australian labradoodle dog breed reveals an excess of the poodle genome
Source: PLoS Genet. 2020 Sep 10;16(9):e1008956. doi: 10.1371/journal.pgen.1008956 (PMC7482835; doi:10.1371/journal.pgen.1008956)
Supplement: S2 Table — Summary of breed abbreviations corresponding to Fig 3A and 3B. (DOCX) [file pgen.1008956.s005.docx]

**S2 Table**. **Breed abbreviations.** Summary of breed abbreviations corresponding to Figs 3A and 3B.

| Abbreviation | Breed |
| --- | --- |
| ACKR | American Cocker Spaniel |
| AESK | American Eskimo Dog |
| AFGH | Afghan Hound |
| AHRT | American Hairless Terrier |
| AIRT | Airedale Terrier |
| AKIT | Akita |
| ALBD | Australian labradoodle |
| AMAL | Alaskan Malamute |
| AMST | American Staffordshire Terrier |
| ANAT | Anatolian Shepherd |
| AUCD | Australian Cattle Dog |
| AUSS | Australian Shepherd |
| AUST | Australian Terrier |
| AZWK | Azawakh |
| BASS | Basset Hound |
| BEAG | Beagle |
| BEDT | Bedlington Terrier |
| BELS | Belgian Sheepdog |
| BERD | Bearded Collie |
| BICH | Bichon Frise |
| BLDH | Bloodhound |
| BMAL | Belgian Malenois |
| BMD | Bernese Mountain Dog |
| BOER | Boerboel |
| BORD | Border Collie |
| BORT | Border Terrier |
| BORZ | Borzoi |
| BOST | Boston Terrier |
| BOUV | Bouvier des Flandres |
| BOX | Boxer |
| BPIC | Berger Picard |
| BRIA | Briard |
| BRIT | Brittany |
| BRTR | Black Russian Terrier |
| BRUS | Brussels Griffon |
| BSJI | Basenji |
| BULD | Bulldog |
| BULM | Bulmastiff |
| BULT | Bull Terrier |
| CAIR | Cairn Terrier |
| CANE | Cane Corso |
| CARD | Cardigan Welsh Corgi |
| CCRT | Curly Coated Retriever |
| CHIH | Chihuahua |
| CHIN | Japanese Chin |
| CHOW | Chow Chow |
| CIRN | Cirneco dell'Etna |
| CKCS | Cavalier King Charles Spaniel |
| COLL | Collie |
| COOK | Chinook |
| COTO | Coton du Tulear |
| CPAT | Cane Paratore |
| CRES | Chinese Crested |
| DACH | Dachshund |
| DALM | Dalmatian |
| DANE | Great Dane |
| DDBX | Dogue de Bordeaux |
| DEER | Scottish Deerhound |
| DOBP | Doberman Pinscher |
| ECKR | English Cocker Spaniel |
| ESET | English Setter |
| ESSP | English Springer Spaniel |
| EURA | Eurasier |
| FBUL | French Bulldog |
| FCR | Flat-coated Retriever |
| FIEL | Field Spaniel |
| FINS | Finish Spitz |
| FOXH | Foxhound |
| GDJK | Golden Jackal |
| GLEN | Glen of Imaal Terrier |
| GOLD | Golden Retriever |
| GORD | Gordon Setter |
| GPYR | Great Pyrenees |
| GREE | Greenland Sledge Dog |
| GREY | Greyhound |
| GSD | German Shepherd Dog |
| GSHP | German Shorthaired Pointer |
| GSMD | Greater Swiss Mountain Dog |
| GSNZ | Giant Schnauzer |
| GWHP | German Wirehaired Pointer |
| HAVA | Havanese |
| HUSK | Siberian Husky |
| IBIZ | Ibizan Hound |
| ICES | Icelandic Sheepdog |
| INCA | Peruvian Hairless dog |
| IRIT | Irish Terrier |
| ISET | Irish Setter |
| ITGY | Italian Greyhound |
| IWOF | Irish Wolfhound |
| IWSP | Irish Water Spaniel |
| JACK | Jack Russell Terrier |
| KEES | Keeshond |
| KELP | Kelpie |
| KERY | Kerry Blue Terrier |
| KOMO | Komondor |
| KUVZ | Kuvasz |
| LAB | Labrador Retriever |
| LBD | Labradoodle |
| LEON | Leonberger |
| LHSA | Lhasa Apso |
| LMUN | Large Munsterlander |
| LVMD | Levriero Meridionale |
| MAAB | Mastino Abruzzese |
| MALT | Maltese |
| MAST | English Mastiff |
| MBLT | Miniature Bull Terrier |
| MNTY | Toy Mnachester Terrier |
| MPIN | Miniature Pinscher |
| MPOO | Poodle - Miniature |
| MSNZ | Miniature Schnauzer |
| MXOL | Xoloitzcuintle - Miniature |
| NEAP | Neapolitan Mastiff |
| NELK | Norwegian Elkhound |
| NEWF | Newfoundland |
| NORF | Norfolk Terrier |
| NOWT | Norwich Terrier |
| NSDT | Nova Scotia Duck Tolling Retriever |
| OES | Old English Sheepdog |
| OTTR | Otter Hound |
| PAPI | Papillon |
| PARS | Parsons Russell Terrier |
| PBGV | Petit Basset Greffon Vendeen |
| PEKE | Pekingese |
| PEMB | Pembroke Welsh Corgi |
| PHAR | Pharoah Hound |
| POM | Pomeranian |
| PTWD | Portuguese Water Dog |
| PUG | Pug Dog |
| PULI | Puli |
| PUMI | Pumi |
| RATT | Rat Terrier |
| REDB | Redbone Coonhound |
| RHOD | Rhodesian Ridgeback |
| ROTT | Rottweiler |
| SALU | Saluki |
| SAMO | Samoyed |
| SCOT | Scottish Terrier |
| SCWT | Soft Coated Wheaten Terrier |
| SHAR | Chinese Shar-pei |
| SHIB | Shiba Inu |
| SHIH | Shih Tzu |
| SILK | Silky Terrier |
| SKIP | Schipperke |
| SLOU | Sloughi |
| SPIN | Spinone Italiano |
| SPOO | Poodle - Standard |
| SSHP | Shetland Sheepdog |
| SSNZ | Standard Schnauzer |
| STAF | Stafforshire Bull Terrier |
| STBD | Saint Bernard |
| SVAL | Swedish Valhund |
| TIBM | Tibetan Mastiff |
| TIBS | Tibetan Spaniel |
| TIBT | Tibetan Terrier |
| TPOO | Poodle - Toy |
| TURV | Belgian Tervuren |
| TYFX | Toy Fox Terrier |
| VIZS | Vizsla |
| VPIN | Volpino Italiano |
| WEIM | Weimaraner |
| WFOX | Wire Fox Terrier |
| WHIP | Whippet |
| WHPG | Wirehaired Pointing Griffon |
| WHWT | West Highland White Terrier |
| WOLF | Grey Wolf |
| XIGO | Xigou |
| XOLO | Xoloitzcuintle |
| YORK | Yorkshire Terrier |
